# Draft Genome Sequence of Anammox Bacterium “Candidatus Scalindua brodae,” Obtained Using Differential Coverage Binning of Sequencing Data from Two Reactor Enrichments

**DOI:** 10.1128/genomeA.01415-14

**Published:** 2015-01-08

**Authors:** Daan R. Speth, Lina Russ, Boran Kartal, Huub J. M. op den Camp, Bas E. Dutilh, Mike S. M. Jetten

**Affiliations:** a Ecological Microbiology, IWWR, Radboud University Nijmegen, Nijmegen, The Netherlands; b Theoretical Biology and Bioinformatics, Utrecht University, Utrecht, The Netherlands; c Centre for Molecular and Biomolecular Informatics, Radboud University Medical Centre, Nijmegen, The Netherlands; d Instituto de Biologia, Universidade Federal do Rio de Janeiro, Rio de Janeiro, Brazil; e Department of Biotechnology, Delft University of Technology, Delft, The Netherlands

## Abstract

We present the draft genome of anammox bacterium “*Candidatus* Scalindua brodae,” which at 282 contigs is a major improvement over the highly fragmented genome assembly of related species “*Ca.* Scalindua profunda” (1,580 contigs) which was previously published.

## GENOME ANNOUNCEMENT

Anammox bacteria are major players in the global nitrogen cycle, capable of anaerobically oxidizing ammonium to dinitrogen gas, using nitrite as the electron acceptor (1). All currently known anammox bacteria form the monophyletic order *Brocadiales* within the phylum *Planctomycetes* (2). Until now, draft genomes of four anammox species have been reported (3–6). The genome assemblies of “*Candidatus* Kuenenia stuttgartiensis” and “*Ca.* Jettenia caeni” are in 5 and 4 contigs, respectively, whereas the draft genome of “*Ca.* Brocadia fulgida” (411 contigs) is fragmented and the “*Ca.* Scalindua profunda” draft genome (1,580 contigs) is highly fragmented.

Despite advances in culturing techniques, no pure culture of anammox bacteria exists. This restricts genome-sequencing efforts to metagenomic sequencing and binning (7, 8). Here we employed a differential coverage binning approach (9) to increase the confidence of the binning result. We combined sequencing data from Russ et al. (10) with sequencing data from the enrichment culture used as seed for the experimental reactor described in Russ et al. (10). The raw sequence data are available in DDBJ/EMBL/Genbank under accession no. ERX443234 and SRX719339. DNA isolation and sequencing of both enrichments was performed as described previously, using the Powersoil DNA isolation kit (Mo-Bio, Carlsbad, CA, USA) according to the manufacturer’s instructions and the Ion Torrent 200 bp workflow (10).

All data were co-assembled using the CLC genomics workbench (v7.0.4, CLCbio, Arhus, Denmark) *de novo* assembler, using word size 35 and bubble size 5,000. The obtained contigs were binned with a workflow modified from Albertsen et al. (9) using custom scripts available at http://www.github.com/dspeth/bioinfo_scripts. The binned “*Ca*. Scalindua brodae” genome consisted of 282 contigs and was annotated using Prokka 1.10 (11) followed by manual curation. Frameshifts were corrected using the CLC genomics workbench (v7.0.4, CLCbio, Arhus, Denmark) and a Perl script available at http://www.github.com/dspeth/bioinfo_scripts. The completeness (>92%) of the draft genome was assessed using CheckM (12). The contigs have a total length of 4.1 Mb, average G+C content of 39.6%, and encode 4,016 genes, 39 tRNAs, and 1 rRNA operon.

Hydrazine is a key intermediate in anammox metabolism and the enzymes involved in its turnover are unique to anammox bacteria. The fusion of subunits B and C of the hydrazine synthase operon (*hzsBC*), earlier reported for “*Ca*. Scalindua profunda” (6), was confirmed and additionally the fused genes seemed to have undergone duplication. The presence of two copies of *hzsBC* prohibited resolving their genomic location without mate pair information. As a result, the hydrazine synthase BC gene is present on a separate contig. The hydrazine dehydrogenase is also present in two copies, but their genomic location could be resolved based on sequence difference.

### Nucleotide sequence accession numbers.

This whole-genome shotgun project has been deposited at DDBJ/EMBL/GenBank under the accession no. JRYO00000000. The version described in this paper is the first version, JRYO01000000.

## References

[1] Kartal B, Maalcke WJ, de Almeida NM, Cirpus I, Gloerich J, Geerts W, Op den Camp HJM, Harhangi HR, Janssen-Megens EM, Francoijs K-J, Stunnenberg HG, Keltjens JT, Jetten MSM, Strous M 2011 Molecular mechanism of anaerobic ammonium oxidation. Nature 479:127–130. doi:10.1038/nature10453. 21964329

[2] Jetten MSM, Op den Camp HJ, Kuenen JG, Strous M 2010 Description of the order Brocadiales, p 596–603. *In* Krieg NR, Staley JT, Hedlund BP, Paster BJ, Ward N, Ludwig W, Whitman WB (ed), Bergey’s manual of systematic bacteriology, vol 4 Springer Verlag, Heidelberg, Germany.

[3] Strous M, Pelletier E, Mangenot S, Rattei T, Lehner A, Taylor MW, Horn M, Daims H, Bartol-Mavel D, Wincker P, Barbe V, Fonknechten N, Vallenet D, Segurens B, Schenowitz-Truong C, Médigue C, Collingro A, Snel B, Dutilh BE, Op den Camp HJM, van der Drift C, Cirpus I, van de Pas-Schoonen KT, Harhangi HR, van Niftrik L, Schmid M, Keltjens J, van de Vossenberg J, Kartal B, Meier H, Frishman D, Huynen MA, Mewes H-W, Weissenbach J, Jetten MSM, Wagner M, Le Paslier D 2006 Deciphering the evolution and metabolism of an anammox bacterium from a community genome. Nature 440:790–794. doi:10.1038/nature04647. 16598256

[4] Hira D, Toh H, Migita CT, Okubo H, Nishiyama T, Hattori M, Furukawa K, Fujii T 2012 Anammox organism KSU-1 expresses a NirK-type copper-containing nitrite reductase instead of a NirS-type with cytochrome *cd*_1_. FEBS Lett 586:1658–1663. doi:10.1016/j.febslet.2012.04.041. 22673575

[5] Ferousi C, Speth DR, Reimann J, Op den Camp HJ, Allen JW, Keltjens JT, Jetten MS 2013 Identification of the type II cytochrome *c* maturation pathway in anammox bacteria by comparative genomics. BMC Microbiol 13:265. doi:10.1186/1471-2180-13-265. 24267221 PMC4222556

[6] van de Vossenberg J, Woebken D, Maalcke WJ, Wessels HJCT, Dutilh BE, Kartal B, Janssen-Megens EM, Roeselers G, Yan J, Speth D, Gloerich J, Geerts W, van der Biezen E, Pluk W, Francoijs K-J, Russ L, Lam P, Malfatti SA, Tringe SG, Haaijer SCM, Op den Camp HJM, Stunnenberg HG, Amann R, Kuypers MMM, Jetten MSM 2013 The metagenome of the marine anammox bacterium “*Candidatus* Scalindua profunda” illustrates the versatility of this globally important nitrogen cycle bacterium. Environ Microbiol 15:1275–1289. doi:10.1111/j.1462-2920.2012.02774.x. 22568606 PMC3655542

[7] van der Star WRL, Miclea AI, van Dongen UGJM, Muyzer G, Picioreanu C, van Loosdrecht MCM 2008 The membrane bioreactor: A novel tool to grow anammox bacteria as free cells. Biotechnol Bioeng 101:286–294. doi:10.1002/bit.21891. 18421799

[8] Sharon I, Banfield JF 2013 Genomes from metagenomics. Science 342:1057–1058. doi:10.1126/science.1247023. 24288324

[9] Albertsen M, Hugenholtz P, Skarshewski A, Nielsen KL, Tyson GW, Nielsen PH 2013 Genome sequences of rare, uncultured bacteria obtained by differential coverage binning of multiple metagenomes. Nat Biotechnol 31:533–538. doi:10.1038/nbt.2579. 23707974

[10] Russ L, Speth DR, Jetten MSM, Op den Camp HJM, Kartal B 2014 Interactions between anaerobic ammonium and sulfur-oxidizing bacteria in a laboratory scale model system. Environ Microbiol 16:3487–3498. doi:10.1111/1462-2920.12487. 24750895

[11] Seemann T 2014 Prokka: rapid prokaryotic genome annotation. Bioinformatics 30:2068–2069. doi:10.1093/bioinformatics/btu153. 24642063

[12] Parks DH, Imelfort M, Skennerton CT, Hugenholtz P, Tyson GW 2014 CheckM: assessing the quality of microbial genomes recovered from isolates, single cells and metagenomes. PeerJ PrePrints 2:e554v1. doi: 10.7287/peerj.preprints.554v1. PMC4484387 25977477

